# Genome-Wide Associative Study of Phenotypic Parameters of the 3D Body Model of Aberdeen Angus Cattle with Multiple Depth Cameras

**DOI:** 10.3390/ani12162128

**Published:** 2022-08-19

**Authors:** Alexey Ruchay, Vladimir Kolpakov, Dianna Kosyan, Elena Rusakova, Konstantin Dorofeev, Hao Guo, Giovanni Ferrari, Andrea Pezzuolo

**Affiliations:** 1 Federal Research Centre of Biological Systems and Agro-Technologies of the Russian Academy of Sciences, 460000 Orenburg, Russia; 2 Department of Mathematics, Chelyabinsk State University, 454001 Chelyabinsk, Russia; 3 College of Land Science and Technology, China Agricultural University, Beijing 100083, China; 4 Department of Land, Environment, Agriculture and Forestry, University of Padova, 35020 Legnaro, Italy

**Keywords:** precision livestock farming, phenotypic features, body condition score, live weight estimation, multiple depth cameras

## Abstract

**Simple Summary:**

This article aims to develop a new approach to the lifetime evaluation of cattle by 3-D visualization of economic-biological and genetic features. The following indicators were selected as phenotypic features: chest width and chest girth retrieved by 3-D model and meat output on the bones. Correlation analysis showed a reliable positive relationship between chest width and meat output on the bones, which can potentially be used for lifetime evaluation of meat productivity of animals. Genome-wide associations analysis revealed the following potential loci of quantitative traits on cattle chromosomes for chest width, chest girth, and meat output on bones.

**Abstract:**

In beef cattle breeding, genome-wide association studies (GWAS) using single nucleotide polymorphisms (SNPs) arrays can reveal many loci of various production traits, such as growth, productivity, and meat quality. With the development of genome sequencing technologies, new opportunities are opening up for more accurate identification of areas associated with these traits. This article aims to develop a novel approach to the lifetime evaluation of cattle by 3-D visualization of economic-biological and genetic features. The purpose of this study was to identify significant variants underlying differences in the qualitative characteristics of meat, using imputed data on the sequence of the entire genome. Samples of biomaterial of young Aberdeen-Angus breed cattle (n = 96) were the material for carrying out genome-wide SNP genotyping. Genotyping was performed using a high-density DNA chip Bovine GPU HD BeadChip (Illumina Inc., San Diego, CA, USA), containing ~150 thousand SNPs. The following indicators were selected as phenotypic features: chest width and chest girth retrieved by 3-D model and meat output on the bones. Correlation analysis showed a reliable positive relationship between chest width and meat output on the bones, which can potentially be used for lifetime evaluation of meat productivity of animals.

## 1. Introduction

Growing population demand for meat and dairy products has led to the rapid growth of animal husbandry worldwide. To increase productivity in animal husbandry, an objective and daily assessment of the productivity of animals in industrial livestock complexes is necessary. Given the complexity of traditional approaches to expert assessment, developing new methods for automatic assessment and forecasting of animal productivity, considering modern technologies in the field of engineering is a significant scientific and practical task [1,2]. This is especially important for predicting the productivity of an animal since this factor determines the effectiveness of this branch of animal husbandry. Conducting population-based genetic and genome-wide association studies to identify candidate genes for economically useful cattle traits is a real problem for farms engaged in raising and fattening cattle. In fact, it is necessary to develop new methods for automatically evaluating and predicting productivity based on external characteristics, to avoid routine work and high costs. These methods can improve the qualitative and quantitative characteristics of animal husbandry. Future technologies should be based on new solutions for 3-D visualization of economic, biological, and genetic characteristics of animals through non-contact measurement of morphological characteristics of the animal, comparison with the presence of SNP markers, and subsequent data processing.

Genome-wide association studies (GWAS) have been a useful tool in medical practice for several years [3,4]. With the development of sequencing technologies, this analysis became available for other industries, so GWAS got its development in animal husbandry [5,6] and crop production [7]. The results of this analysis can provide helpful information about the genetic architecture of the organism, as well as identify potential areas of the genome associated with quantitative traits (QTL) and evaluate their implementation in the phenotype. Currently, GWAS is used to identify single nucleotide polymorphisms (SNP) candidate genes of many economically useful traits in livestock [8,9,10]. Thus, in dairy cattle, a high quantity of loci in the genome is associated with quantitative traits (QTL), which was confirmed by using microchips with a panel density of 50,000 SNP or more [11,12]. Similar studies are conducted for meat breeds, but they only concern signs of meat productivity, particularly tenderness and marbling. For these reasons, GWAS in agriculture is a powerful tool that can identify new genetic variants of traits that affect the phenotype of an animal, especially the exterior and meat qualities [13].

Aberdeen Angus is considered one of the best breeds among hundreds of others for marbled beef production, characterized by an ideal structure, excellent taste, and pleasant aroma. The meat of the Aberdeen Angus breed, along with other meat breeds, is considered the best for food purposes [14]. Currently, several data from several genome-wide associative studies have been published on Aberdeen Angus cattle concerning such signs as the composition of fatty acids, the structure of mammary glands, pregnancy, and iron concentration in the longest back muscle [15,16]. However, the phenotypic parameters of the exterior of Aberdeen-Angus cattle remain poorly studied. Similarly, a small number of published studies used calculated genome-wide sequence (WGS) data for these indicators.

In this regard, the purpose of this study was to analyze the genome structure of an experimental group of young cattle of the Aberdeen-Angus breed based on GWAS phenotypic parameters of the exterior.

The main contributions of this article are as follows:

- Computer vision can provide the lifetime evaluation of cattle by 3-D visualization of economic-biological and genetic features.
- A test analysis of genome-wide associations revealed the potential loci of quantitative traits on cattle chromosomes for chest width, chest girth, and meat output on bones.
- Database creation: the database contains raw RGB-D images, point clouds, and live weight, measurements obtained from a 3-D model (live weight, withers height, hip height, chest width, chest height), genotyping data, slaughter characteristics—live weight, meat yield, stress losses, characteristics of the front quarters (19 parameters), characteristics of the rear quarters (15 parameters), characteristics of offal (22 parameters) for 96 Aberdeen-Angus cattle.

The study offers an innovative approach to developing technology for assessing and predicting animal productivity based on automated body measurements (live weight, withers height, hip height, chest width, chest height) using a 3-D model of Aberdeen Angus cattle obtained from multiple depth cameras. The proposed technology is based on methods of non-contact 3-D reconstruction of the animal model, productivity parameters, genetic characteristics, and animal health, that are closely related to the features of their constitution and exterior.

## 2. Literature Review

Currently, DNA technologies are widely used to develop methods for managing the flow of genetic material and conducting genetic monitoring to assess the degree of polymorphism. Progress in the development of genetic research is due to the presence of informative genetic markers. In the last decade, a trend has developed in world science called Marker assisted Selection (MAS) [17,18]. Research on this issue is actively conducted to identify genes (individual loci) responsible for a particular desirable trait [19,20,21]. Currently, using molecular biology methods, information about genetic markers, and their relationship with economic and useful traits, it is possible to conduct the selection process at a qualitatively new level. For example, using advanced technologies, markers were found that are uniquely associated with milk productivity (the Kappa-casein milk gene and the growth hormone somatotropin gene), immune resistance (the BolaDRB3 gene), meat tenderness (the CAPN1, CAST, TG5 genes), and meat marbling (Lep, TG5, DGAT1) in cattle.

Many factors influence the efficiency of animal production; one of the most significant is the genetic potential of animals [22,23]. The use of informative DNA marker data allows selection at an early age and also characterizes the polygenic nature of inheritance [24,25].

Thus, genotyping of herds by individual DNA markers makes it possible to create groups of animals that differ in productivity potential, quality composition of products, level and direction of metabolism, and energy in the animal body [26].

For the study of beef cattle, the main task is to control the development of muscles and, as a result, the quality of meat. Beef quality is a complex variable phenotype detected only after slaughter [27,28]. To date, the identification of relevant genetic and genomic markers continues, especially for determining the tenderness of the meat. Therefore, the authors proposed strategic stages for studying marker expression based on improving the sensory quality of beef, starting with the detection of biomarkers that identify the primary biological properties of meat [29,30].

The emergence of genomic selection with correspondingly improved accuracy prediction will accelerate the introduction of elite genetics in enhancing breeding efficiency in beef cattle populations. The existing problems associated with this approach can be overcome long-term by increasing international collaborative efforts. Still, in the short term, they will not eliminate the continuing need for accurate measurement of the primary phenotype [31,32].

## 3. Material and Methods

### 3.1. Animals and Ethics

The young cattle of the Aberdeen-Angus breed (n = 96) were selected as the object of the study. At the time of the experiment, the age of the animals was 16.5 months; the live weight was 614.9 kg. All experimental procedures and ethical standards were approved by the Committee for Control over the Maintenance and Use of Laboratory Animals in accordance with the “Policy of the Scientific Center for Work with Laboratory Animals” of the Federal State Budgetary Scientific Institution “Federal Research Centre of Biological Systems and Agrotechnologies of the Russian academy of sciences”. Extracts from the minutes of the meeting of the Commission for control over the maintenance and use of laboratory animals No. 4 dated 17 January 2021 were received on 20 March 2022. During the research, measures were taken to ensure a minimum of animal suffering and to reduce the number of experimental samples studied.

The animals were kept in a separate group at a feedlot in the Ramonsky district of the Voronezh Region, Russia. The animals were of the same sex and the same age. The feeding and maintenance conditions were the same for the whole group. Upon completion of fattening, the animals were slaughtered at a commercial slaughterhouse owned by a meat processing plant. Animals were identified using an RFID chip, and carcasses were identified using tags and cooled for 24–48 h after slaughter. After cooling, the carcass was cut into marketable pieces.

The morphological characteristics of the animals were measured in accordance with the guidelines issued by the Committee. Data collection in this study was carried out without restriction on cows. Therefore, this study does not require the approval of the Animal Care and Use Committee, which eliminates the need for the approval of environmental monitoring or animal sciences.

TA database of phenotypic traits of Aberdeen-Angus bulls was formed to conduct genome-wide associative studies. The indicators were divided into external ones—height at the withers and in the sacrum, chest width behind the shoulder blades, chest girth, and meat output on the bones.

Measurements of exterior features and meat productivity were made using a system of accurate measurement of the body of live cattle using three-depth chambers and non-rigid three-dimensional shape restoration [33,34,35].

### 3.2. Database

A database containing the following data for each of the 96 animals was collected: RFID chip number, RGB images, depth maps, and point clouds; live weight, linear measurements obtained from a 3D model (live weight, withers height, hip height, chest width, chest height), genotyping data, slaughter characteristics—live weight, meat yield, stress losses, characteristics of the front quarters (19 parameters), characteristics of the rear quarters (15 parameters), characteristics of offal (22 parameters). This database is publicly available [36].

### 3.3. Genotyping

Genomic DNA was extracted from the whole blood using «DNA-Extran» (Syntol LLC, Moscow, Russia). The concentration of genomic DNA was detected by Qubit (Thermofisher, Waltham, MA, USA), and the quantity of DNA was estimated by Nanodrop ND-1000 (ThermoFisher, Waltham, MA, USA). The 96 animals were genotyped with the Illumina BovineHD Beadchip (HD; 141,716 markers) following standard procedures of the manufacturer.

The unmapped SNPs and SNPs present on X and Y chromosomes and mitochondrial DNA were removed. SNPs located on autosomes were put to analysis. The quality control procedure was carried out using PLINK [37]. The Plink v. 1.9. software was used for quality control of genotyped breeds based on the following parameters: call-rate for all studied SNPs for an individual sample is not lower than 90% (--reason); Call-rate for each of the studied SNPs for all genotyped samples is not lower than 90% (--Geno); frequency of occurrence of minor alleles (DMRV) 0.05 (0.05--DMRV); deviation of SNP genotypes from the Hardy distribution-Weinberg in the aggregate of tested samples with confidence *p*-value < 10–6 (--rhb). 

Additionally, a heterozygosity test was conducted to determine which samples deviate from the average value by more than 3 standard deviations to exclude them from the analysis. Finally, the principal component method was used to estimate the population stratification, Principal component analysis (PCA) [38].

The minor allele frequency (MAF) is the frequency of the least common allele for that SNP in the population. The MAF for each autosome and the overall mean MAF in cattle breed were estimated by using PLINK. The significance of breed differences in MAF was done using the SPSS package. The MAF values of all the SNPs were classified into four different categories viz. monomorphic/fixed SNPs (MAF ¼ 0), rare SNPs (MAF > 0–< 0.05), intermediate SNPs (MAF ≥ 0.05–< 0.1), and common SNPs (MAF ≥ 0.1–≤ 0.5).

Multiple linear regression analysis implemented in Plink 1.90 was used to identify associations of SNP markers with the studied phenotypic indicators. GWAS was performed for the six morphometric traits using PLINK [37]. A linear regression using an additive genetic model was applied and defined as follows:

y = Xb+ Wg + e,(1)

where y was a vector of morphometric traits; b was a vector of fixed effects including Live weight, Withers height, Hip height, Chest width, Chest girth, and Meat output on bones and linear discriminant functions; g was a vector for the SNP effects; e was a vector of random residual effects with e~N(0, $I\sigma_{e}{}^{2}$); and X, W were incidence matrices for b and g, respectively.

Since many hypotheses are tested simultaneously during the SWOT analysis (141,716 SNPs were tested in our study), the correction was made for multiple hypothesis testing. In this study, the Bonferroni correction was applied, and the threshold for the *p*-value in GWAS was calculated using the formula:

*p* ≤ α/m (2)

where α = 0.05, m = 111,158 SNP. 

Thus, during the GWAS, associations with *p* < 4.5 × 10^−7^ were considered statistically reliable. Data visualization was carried out in the qqman package using the R programming language [39].

### 3.4. RGB-D Image Capture System

The data collection unit was installed in the passage to the hall with a feed system. All measurements were carried out on a walking animal from three points of view since it is impossible to require the animal to stop and remain motionless. Two RGB-D cameras were located to the right and left of the animal aisle at a distance of about 2.0 m; the third Kinect camera was located above the aisle at a height of about 3.0 m above the ground. The installation used three identical Microsoft Kinect v2 cameras (Microsoft, Redmond, WA, USA) that receive RGB and depth images from the left, right, and upper sides of the animal. Each depth camera was connected to a laptop, and all laptops were connected to a local network. Synchronously captured images in RGB-D format were recorded on the corresponding laptop for each camera. Data collection and storage were implemented based on Software Development Kit 2.0 (SDK). Each camera initialized by the start signal starts capturing frames at 30 Hz. The time on the laptops was synchronized, and the best match of the point cloud could be selected within the shortest time intervals between the three devices. Transformation matrices using external calibration were calculated distances between different cameras to align the point cloud. The resolutions of RGB images and depth images are 1920 × 1080 and 512 × 424 pixels, respectively. More information can be found in [33].

## 4. Results

### 4.1. RGB-D Image Capture System

**Figure 1:**
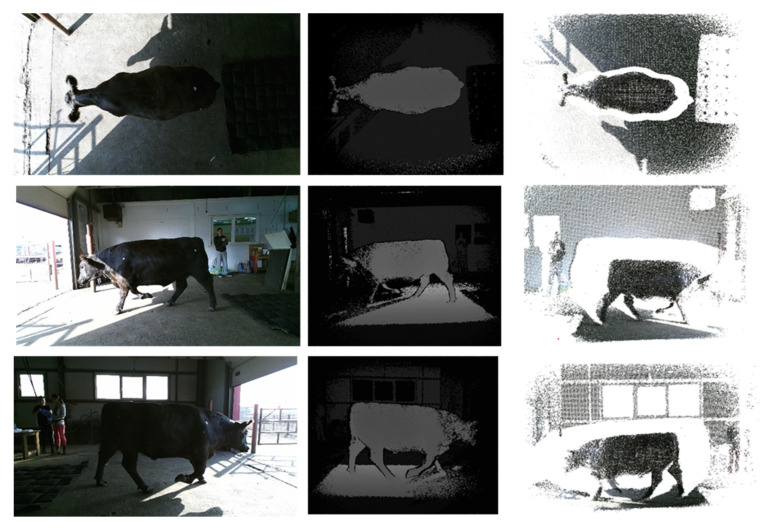
RGB images, depth maps, and point clouds of the cattle captured with three Kinect cameras.

The point cloud fusion and 3-D reconstruction of live cattle were carried out using the proposed automated computer vision system [33] that can generate one accurate 3D model of live cattle based on RGB-D data. Figure 1 shows RGB images, depth maps, and point clouds of the cattle captured by three Kinect cameras.

**Figure 2:**
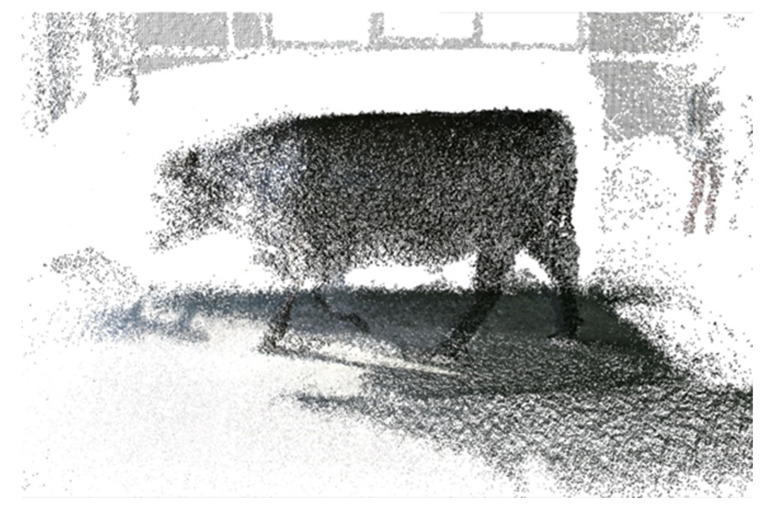
3-D point cloud model of the cattle using the proposed system for 3-D reconstruction.

Finally, the point cloud fusion was elaborated to generate the 3-D cattle model. Figure 2 shows a 3-D reconstruction of the cattle obtained in the proposed system [33]. The 3-D cattle models are accurate and consistent with the actual figure of the animal.

The estimated body measurements were according to the following definitions:

- withers height is defined as the height of the highest point at the withers;
- hip height in the sacrum is the height of the highest point at the sacrum;
- chest width is defined as the width vertically tangent to the posterior corner of the shoulder blade;
- chest girth is defined as the vertical tangent to the back corner of the shoulder blade.

**Figure 3:**
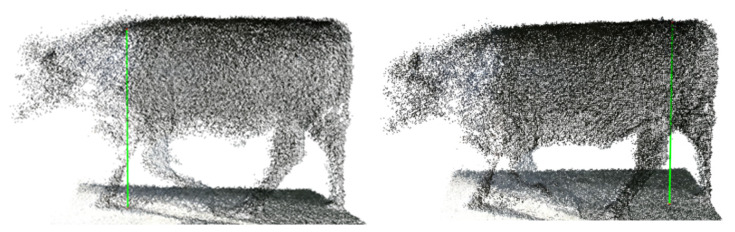
Measurement of withers height and hip height on a 3-D animal model.

**Figure 4:**
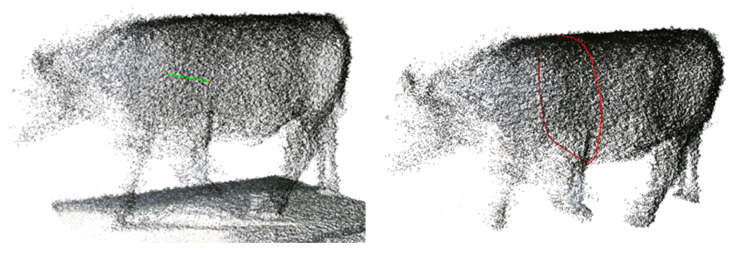
Measurement of chest width and chest girth on a 3-D animal model.

The proposed 3-D animal automatic measurement system was implemented using three RGB-D cameras [33]. With a 90% confidence level, measurement errors in the proposed system are less than 3%. To measure linear parameters such as the withers height and hip height, we calculated the Euclidean distance from the corresponding landmark on the point cloud to the ground plane. Similarly, chest width could be measured as the distances between the corresponding landmarks on the point cloud. The heart girth is the perimeter around the heart on the point cloud. First, a plane defined by the two Y and Z coordinate axes and passing through the landmark is created. Then, a convex hull for the intersection points between the plane and the point cloud is constructed using PCL. The perimeter curve consists of connected points of the hull. Experimental results show that the proposed approach can serve as a new accurate method for the non-contact body measurement of cattle. Figure 3 and Figure 4 show examples of each measurement for one animal.

The obtained 3-D models of animal bodies made it possible to collect measurements of live weight, withers height, hip height, chest width, and chest girth. In addition, the process of cutting carcasses at the meat processing plant provided valuable data on meat output on bones. All the listed data are presented in Table 1.

Based on these results, indicators characterized by multiple gene action were selected for testing genome-wide associative studies.

The created database is open and accessible to the research community [36]. The dataset contains synchronized raw depth maps, RGB images, restored point clouds from left, right, and top views (the sizes of depth and RGB images are 512 × 424 and 1920 × 1080 pixels, respectively), transformation matrices, and aligned point clouds for 96 cattle.

### 4.2. GWAS Analysis

At the initial stage, the relationship between the main features of the exterior was evaluated. The results showed that the live weight, withers height, and rump of the animal had a weak and negative relationship with meat output on the bones (r = −0.037, …, −0.096), which indicated the absence of a reliable relationship. At the same time, such signs as chest width and chest girth were significantly correlated with meat output, respectively, r = 0.279 (*p* < 0.001) and r = 0.100. These parameters were selected for further analysis.

**Figure 5:**
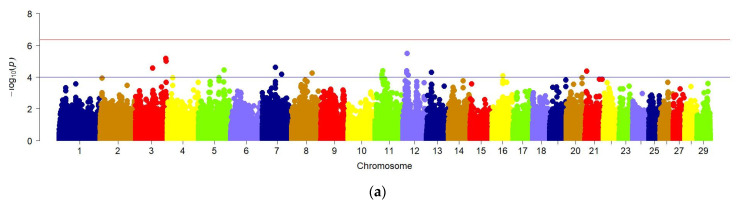

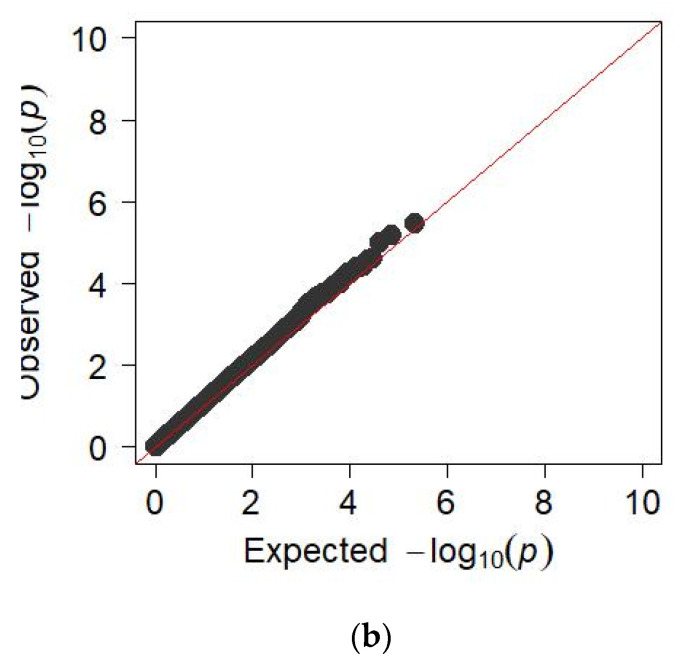
(**a**) Manhattan plots of WGS for chest width with significance thresholds indicated at −log_10_*p* > 4.5 × 10^−7^; Panel (**a**) shows the chromosome regions associated with chest width, using ~150,000 imputed sequence SNPs. (**b**) The quantile-quantile (QQ) plots for the studied quality trait. Panels (**b**) have shown the QQ plots for the chest width trait.

GWAS for chest width trait did not reveal significant regions in the genome of the studied animals, which is demonstrated in the Manhattan graph (Figure 5a). Therefore, a quantile-quantile graph (QQ) of the distribution of all *p*-values obtained during the analysis of each feature compared to the expected *p*-values was constructed to verify the results obtained. When checking the GWAS, the absence of a relationship between most of the tested SNPs and the trait under study was assumed, so they must correspond to a uniform distribution. In this case, the QQ graph for the chest width of the animal does not show a clear deviation from the normal distribution of several values, which may indicate the absence of a true relationship of SNP with this trait (Figure 5b).

**Figure 6:**
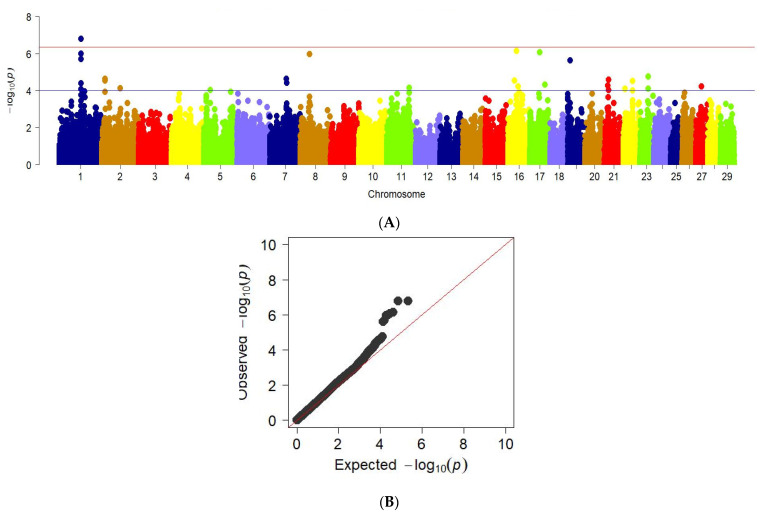
Manhattan plots of WGS for chest girth with significance thresholds indicated at −log_10_*p* > 4.5 × 10^−7^. B The quantile-quantile (QQ) plots for the studied quality trait. Panel (**A**) shows the chromosome regions associated with chest girth using ~150,000 imputed sequence SNPs. The panels (**B**) have shown the QQ plots for the chest girth trait.

GWAS for chest girth traits revealed significant regions in the genome of the studied animals, in particular in chromosomes 1, 2, 7, 8, 16, 17, 19, 21, 22, 23, and 27. This is shown in the Manhattan graph (Figure 6a). In addition, the QQ graph for the chest girth trait confirms a deviation from the normal distribution of several values, which may indicate a true relationship of SNP with this trait (Figure 6b).

**Figure 7:**
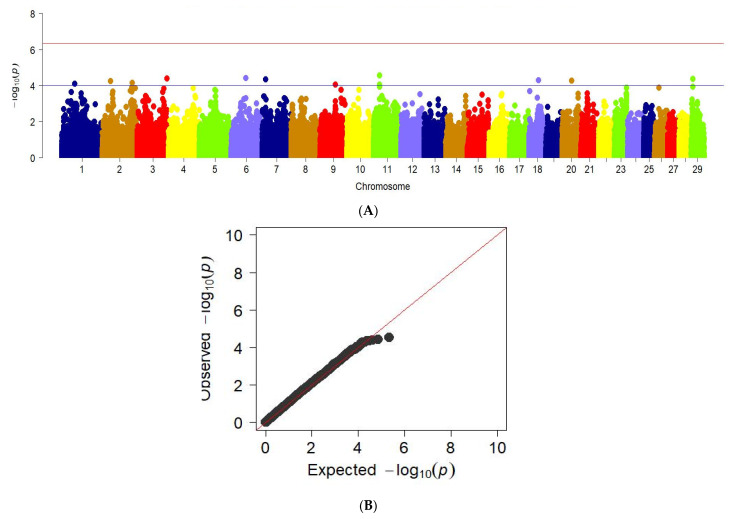
Manhattan plots of WGS for meat output on bones with significance thresholds indicated at −log_10_*p* > 4.5 × 10^−7^. B The quantile-quantile (QQ) plots for the studied quality trait. Panel (**A**) shows the chromosome regions associated with bone meat output using ~150,000 imputed sequence SNPs. The panels (**B**) have shown the QQ plots for meat output on bones trait.

Analysis of genome-wide associations with ball output demonstrates the following regions in chromosomes 1, 2, 3, 6, 7, 9, 11, 18, 20, and 29. This is shown in the Manhattan graph (Figure 7a). The data is confirmed by the QQ graph for this feature.

## 5. Discussion

The discovery of single nucleotide polymorphism (SNP) and subsequent genotyping of a large number of animals made it possible to conduct a large-scale analysis to understand the biological processes underlying the variability of animal populations. The availability of SNP panels for the entire genome made it possible to implement genomic prediction in many livestock species [40,41]. Unlike traditional quantitative trait locus mapping strategies, GWAS opens up new opportunities for the effective use of mongrel cattle populations for high-resolution loci mapping with even modest effects underlying important productive traits [42]. In addition, developing sequencing technologies provides new opportunities for improving the accurate mapping of quantitative trait loci. Given the relatively high cost of sequencing, there is a tendency for genotyped animals (mainly genotyped by SNP density from 10 to 700 thousand) to be accurately calculated to the genome-wide sequence (WGS). The result obtained during GWAS has greater power and accuracy for detecting a significant number of quantitative trait loci [9,43].

During the work, it was found that the indicators of meat cuts had a higher variability relative to the yield of offal and exterior parameters.

In the series of observations, correlation analysis showed a reliable positive relationship between chest width and meat output on the bones, which can potentially be used for lifetime evaluation of meat productivity of animals. Data regarding the results of multidimensional scaling are similar to those provided by the literature [44], which established the heterogeneity of the sample by the level of genetic variation, possibly due to genetic drift [45].

The results obtained using GWAS showed that the resulting additive genetic variance is unevenly distributed across the genome. Moreover, some regions explain a larger share compared to others. GWAS identified the following potential loci of quantitative traits on cattle chromosomes for: live weight—BTA1, 3, 5, 15, 16, 17, 19, 20, 23, and 29; withers height—BTA2, 3, 5, 6, 7, 10, 12, 14, 15, 21, 22, 24, 26, and 29; hip height—BTA2, 3, 5, 6, 8, 9, 10, 12, 20, 21, 26, and 29; chest width—2, 3, 5, 7, 8, 11, 12, 13, 16, 20, and 21; chest girth—BTA1, 2, 5, 7, 8, 11, 16, 17, 19, 21, 22, and 23; meat output on bones—BTA1, 2, 3, 5, 6, 7, 9, 11, 18, 20, and 29, which is partially consistent with the data of [45,46,47,48,49].

## 6. Conclusions

In this paper, the genetic parameters of phenotypic features of the exterior 3-D animal model of the Aberdeen-Angus breed were evaluated by the method of genome-wide associative research (GWAS). GWAS provides valuable estimates of genetic parameters and key information about the regions underlying the variability of the traits discussed. Correlation analysis showed a positive, reliable relationship between chest width and meat output on the bones, which can potentially be used for lifetime evaluation of meat productivity of animals.

The important regions identified in this study can provide valuable biological information to improve the accuracy of genomic selection in breeding programs for Aberdeen Angus beef cattle. These studies are planned to be applied to other cattle breeds. The main motivation for conducting genome-wide association studies (GWAS) in domestic animals, such as beef cattle, is the detection of genes or causal mutations that contribute to the phenotype of economically important traits. Such results may be important for improving the breeding value assessment and our understanding of the mechanisms underlying the long-term selection response in the artificial breeding program [13]. 

## Figures and Tables

**Table 1:** Characteristics of exterior features, fattening, and meat productivity of a sample of Aberdeen-Angus breed animals.

Indication	X	m	Cv, %	Min	Max
Live weight, kg	614.9	3.4	5.4	556.0	746.0
Withers height, cm	143.1	0.6	3.9	119.0	153.0
Hip height, cm	146.4	0.6	3.9	122.0	156.0
Chest width, cm	52.2	0.2	3.5	48.0	56.0
Chest girth, cm	217.1	0.6	2.5	205.0	230.0
Meat output on bones, %	60.4	0.1	2.1	57.0	63.3

## Data Availability

The data presented in this study are available.
